# Development of an All-Marine 3D Printed Bioactive Hydrogel Dressing for Treatment of Hard-to-Heal Wounds

**DOI:** 10.3390/polym15122627

**Published:** 2023-06-09

**Authors:** Patrik Stenlund, Linnea Enstedt, Karin Margaretha Gilljam, Simon Standoft, Astrid Ahlinder, Maria Lundin Johnson, Henrik Lund, Anna Millqvist Fureby, Mattias Berglin

**Affiliations:** 1 Department of Methodology, Textile and Medical Technology, RISE Research Institutes of Sweden AB, Arvid Wallgrens backe 20, SE-413 46 Gothenburg, Sweden; 2 Department of Chemical Process and Pharmaceutical Development, RISE Research Institutes of Sweden AB, Drottning Kristinas väg 61B, SE-114 28 Stockholm, Swedenmaria.lundinjohnson@gmail.com (M.L.J.);; 3 Regenics AS, Gaustadalléen 21, N-0349 Oslo, Norway; 4 Department of Agriculture and Food, RISE Research Institutes of Sweden AB, Frans Perssons väg 6, SE-412 76 Gothenburg, Sweden; astrid.ahlinder@ri.se; 5 Department of Chemistry and Molecular Biology, University of Gothenburg, Kemigården 4, SE-412 96 Gothenburg, Sweden

**Keywords:** biomaterial, 3D printed scaffolds, wound dressing, alginate, nanocellulose, salmon roe, hard-to-heal wounds

## Abstract

Current standard wound care involves dressings that provide moisture and protection; however, dressings providing active healing are still scarce and expensive. We aimed to develop an ecologically sustainable 3D printed bioactive hydrogel-based topical wound dressing targeting healing of hard-to-heal wounds, such as chronic or burn wounds, which are low on exudate. To this end, we developed a formulation composed of renewable marine components; purified extract from unfertilized salmon roe (heat-treated X, HTX), alginate from brown seaweed, and nanocellulose from tunicates. HTX is believed to facilitate the wound healing process. The components were successfully formulated into a 3D printable ink that was used to create a hydrogel lattice structure. The 3D printed hydrogel showed a HTX release profile enhancing pro-collagen I alpha 1 production in cell culture with potential of promoting wound closure rates. The dressing has recently been tested on burn wounds in Göttingen minipigs and shows accelerated wound closure and reduced inflammation. This paper describes the dressings development, mechanical properties, bioactivity, and safety.

## 1. Introduction

The development of advanced and effective wound dressings is a critical area of research in modern medicine, particularly for hard-to-heal wounds, which can cause significant physical and psychological distress to patients. There is an urgent need for novel and innovative dressings that can not only cover and protect the wound while creating a moist environment, but which also actively support tissue regeneration and suppress inflammation.

Wound healing starts immediately upon an injury and involves inflammation, cell proliferation, and tissue remodeling [1]. It is an intricate process requiring a balanced interplay of cellular functions where the early events will dictate the outcome. For example, extended inflammation has been shown to cause excessive scarring [2]. In chronic wounds, this balance is disrupted, resulting in persistent inflammation, impaired angiogenesis and re-epithelialization, and increased protease activity, which lead to delayed or insufficient healing [3,4]. In severe burn wounds, the trauma causes excessive inflammation leading to secondary necrosis and wound progression, which causes delayed healing and excessive scarring [5]. As a key component of the extracellular matrix, collagen plays a central role in the regulation of the wound healing phases serving, for example, as a scaffold for cellular attachment and function [3]. The burden of wounds on healthcare systems is sharply increasing due to an aging population and rising cases of diabetes and obesity. One of the most serious complications of diabetes is the development of chronic wounds, such as diabetic foot ulcers. Both burns and chronic wounds are hard-to-heal and may result in prolonged hospital stay, patient discomfort, and massive scarring. In addition, the patients may suffer from serious complications including sepsis and amputations [6,7]. Current standard of care for burns and chronic wounds serve to cover the wound, protect from infection, and provide moisture. The present gold standard in wound healing is silver-containing hydrofiber dressings. However, recent findings suggest that silver ion halts wound healing through cytotoxicity [8]. Notably, the increased microbial silver resistance also decreases the efficacy of the dressings to prevent infection [9]. Extensive efforts are devoted to finding improved treatment options for these wounds, and one area that is growing rapidly is the creation of functional scaffolds loaded with biologically active molecules to be delivered at the wound site to support the normal wound healing process [4].

In the search for naturally sourced substances with potential to advance medical applications, salmon roe has been identified as an interesting candidate. HTX™ is the brand name of a mildly processed, sterilized extract from unfertilized salmon roe (*Salmo salar*), anticipated to have beneficial properties for the wound healing process. Salmon roe contains mainly proteins, fats, vitamins, and minerals, with zinc being the most prevalent mineral, and Omega-3 and Omega-9 the most predominant fatty acids, according to the NDSR Food and Nutrient Database [10,11]. Originating from unfertilized salmon roe, HTX contains high amounts (about 100 mg/mL) of proteins including the yolk protein Vitellogenin. Vitellogenin is a glycoprotein which provides protein-rich nutrients for developing embryos, has been shown to participate in host immune defense and possess antioxidant properties [12]. Zinc is a fundamental trace element in the body and essential for the normal function of the immune system. The mineral plays an important role for enzymes that regulate cellular signaling pathways and processes. Moreover, it possesses antioxidant and anti-inflammatory properties, even though the underlying mechanisms have not yet been fully identified [13,14]. Omega-3 and Omega-9 have a well-documented moisturizing effect, and they are both frequently used in skin care [15,16]. Omega-3, Omega-6, and Omega-9 acids have been extensively studied and shown to promote wound healing, in part by modulating the inflammatory reaction in wounds [17,18]. Products from cod skin and haruan/snakehead roe have shown anti-inflammatory properties in topical wound treatment [19,20].

The aim of this work was to formulate the HTX with other biocompatible marine components to develop an ecologically sustainable hydrogel-based wound dressing able to release the bioactive HTX into the wound and thereby stimulate healing. The intended mode of action for the hydrogel was to cover and protect the wound while providing a moist and absorbent wound healing environment, with an ancillary effect of supporting wound closure.

The moisture balance plays a major role in facilitating the wound healing process. Moist conditions have been reported to enhance cellular migration, keratinocyte and fibroblast proliferation, angiogenesis, removal of fibrin and dead tissue debris, and collagen synthesis, in contrast to dry conditions [21]. However, for highly exudating wounds, dressings that absorb the excessive fluid are recommended, i.e., a moisture balance should be pursued [22]. A moist environment is also a prerequisite when aiming to deliver bioactive molecules to the wound [21].

Three-dimensional printing is an emerging technology within pharmaceutical science and regenerative medicine, including wound care applications [23,24]. Recent advances in 3D printing technology have led to the development of new and innovative materials, scaffold designs, and wound dressings. The technology allows for the creation of hierarchical structures with high precision and freedom of design, and it is an automated process, which enables high reproducibility [25]. Another desirable aspect is that several materials, cells, and/or bioactive agents can be combined in the ink. As a result, three-dimensional scaffolds that mimic the extracellular matrix environment in the skin can be created, to further facilitate cell proliferation, migration, and differentiation [26].

Alginate is a well-established material used in both bioinks and wound dressings that deserves more attention and further research, specifically for treatment of chronic wounds [27,28]. It is a naturally sourced biopolymer or polysaccharide derived from, for example, brown algae. The biopolymer consists of repeating units of D-mannuronic and L-guluronic acid. Alginate hydrogels can be formed by ionic cross-linking, typically with calcium ions, where the mechanical properties of the hydrogel depend on the alginate concentration, molecular weight, the mannuronic/guluronic stoichiometric ratio, and the calcium concentration, leading to different degrees of cross-linking. As a biopolymer, alginate hydrogels possess desirable properties such as water absorption, rapid gelation, biocompatibility, affordability, biodegradability, and renewability. Notably, dry alginate dressings will absorb wound exudate and are suitable for draining wounds, while it in the hydrogel format is a good choice for dry, necrotic wounds [22,29]. This gives alginate dressings the capacity to maintain a moist microenvironment [30]. Alginate is generally recognized as a biocompatible encapsulation matrix, in which proteins retain their activity. For example, alginate microparticles have been successfully used to encapsulate enzymes and other proteins, showing controlled release properties [30,31].

Nanocellulose can be obtained from renewable sources; in this study it was derived from tunicates (*Ciona intestinalis*), a marine source. Similar to alginate, nanocellulose-based gels and hydrogels support a high water content providing a moist environment for wounds, with the capability to effectively absorb wound exudate. In addition, they meet the expectations of functional wound dressing materials, being non-toxic, and their high hydrophilicity makes them suitable for incorporation of bioactive molecules when rapid release is required [32,33]. Nanocellulose-based scaffolds have shown potential to support regenerative responses resulting in a natural cellular development [34]. In addition to supporting proliferation of cells, nanocellulose possess beneficial rheological properties such as shear thinning and thixotropy. These properties facilitate extrusion and rapid recovery of bioinks, which reduces the risk of cell death and enhances the structural fidelity during printing [35]. Nanocellulose has a high aspect ratio between length and width, this results in a low concentration being needed before physical entanglement of the macromolecule occurs; concentrations below 2.5% have been suggested for bioinks to aid the shape stability [35]. Inks based on a mixture of nanocellulose and alginate have shown to be a modular system for 3D-printing of porous structures where printability and fidelity can be tuned based on the ink composition [36]. The addition of nanocellulose adds stability to the ink whilst printing, and the crosslinked alginate provides a stable crosslinked network over time. The shear thinning ability of both alginate and nanocellulose aids the extrusion of the ink through the syringe. 

This work combines renewable, all-marine-sourced products to create a 3D printed wound dressing, referred to as Collex™, developed for the healing of hard-to-heal wounds, such as chronic wounds and burns, which are both low on exudate. The product developed herein was evaluated in parallel on porcine burn wounds, which indicated that Collex is a potent dressing for the treatment of burn wounds [37]. The work presented herein describes the ink optimization process and characterization of the material properties of this new wound healing dressing, which includes in vitro assays to determine cytotoxicity, the release profile of HTX, and its effect on human fibroblasts.

## 2. Materials and Methods

### 2.1. Ink Formulation and Optimization

The different inks were formulated by mixing medical grade TUNICELL ETC + M, enzymatically pretreated cellulose nanofibrils (E-CNF) supplemented with 4.6% mannitol (OceanTunicell AS, Bergen , Norway), H_2_O, and 0.9% NaCl (Fresenius Kabi AG, Bad Homburg, Germany). Then, sodium alginate (10% *w*/*v* in saline, PRONOVA UP MVG (NovaMatrix DuPont Nutrition Norge AS, Sandvika, Norway) with Mw > 200 kDa and a mannuronic/guluronic (M/G) ratio of ≥1.5, was added and after mixing left to equilibrate for 2 h before adding HTX 12% (*v*/*v*), a purified salmon roe extract (Regenics AS, Oslo, Norway), and mixed once more. All mixing steps were performed by hand for 5 min and the ink was then centrifuged to release air bubbles. The ink composition was systematically varied, as in Table 1. The ink properties were evaluated based on rheology, printability, shape fidelity, handling, and protein release profiles. To better understand and model the responses the experiments were planned according to a full factorial design (R1–R4) with R5 the center point. The data was evaluated in Statistica ver. 14.0.0.15 (StatSoft Europe GmbH, Hamburg, Germany) using the design of experiments (DOE) module.

### 2.2. Ink Rheology

The characterization of the flow behavior and viscoelastic properties of the different inks were conducted using DHR-30 rheometer (TA instruments, New Castle, DE, USA) using a 20 mm upper plate together with a lower Peltier Plate. All rheological characterization were made at 20 °C and the gap used was 500 µm for all experiments. The software TRIOS version #5.5.0.323 (TA instruments, New Castle, DE, USA) was used to process the data, and for each test, 10 points per decade were taken. A 30 s soak time was used to let the sample relax on the plate before the tests were conducted. Three replicates were made of the ink compositions and the reported data was mean ± standard deviation in the graphs.

The flow behavior with respect to shear was determined with a flow sweep between 100 s^−1^ and 0.1 s^−1^ using steady state sensing with maximum of 10 s equilibration time and 10 s sample period using a 5% tolerance to be consecutive within 3 measurements. The stress from the flow sweep was fitted to the Herschel–Bulkley model using the software TRIOS version #5.5.0.323 (TA instruments, New Castle, DE, USA). The Herschel–Bulkley model parameter fit is shown as mean ± standard error (SE). The viscoelastic properties with regards to frequency were characterized using an oscillatory frequency sweep between 0.1 and 30 Hz, within the linear viscoelastic region.

### 2.3. Three-Dimensional Printing of Hydrogels

Inks were prepared by mixing alginate, nanocellulose, saline, and HTX by the ratios given in Table 1. Ink patches, aiming for 200 mg of ink, were 3D printed using a 4th Gen 3D-Bioplotter (EnvisionTEC, Gladbeck, Germany) in an interlaced 90-degree continuous grid pattern composed of 4 layers measuring 16 mm × 16 mm. The internal grid was defined by a lateral filament separation of 1.8 mm, and 0.7 mm from the contour using a 22-gauge sized nozzle and printing pressures and speeds ranging from 0.1–0.4 bar and 4–40 mm/s, respectively. Once the 4 layers were printed, the patch was imaged by the 3D-Bioplotter —sticking nine images together and the result used to evaluate the printability. Thereafter, ink patches were crosslinked in 0.02 M CaCl_2_ solution (Sigma-Aldrich, Darmstadt, Germany) supplemented with HTX (12% *v*/*v*) for at least 30 min. Meanwhile, the ink patches were kept cool and shielded from light by an aluminum foil cover until transferred to an ethanol washed polyethylene/aluminum sachet with an argon atmosphere for storage in the fridge prior to analysis. In experiments including hydrogel control patches, the HTX in both the ink and the crosslinking solution was replaced by 0.9% NaCl. To estimate the patch height, the final construct was transferred and imaged once more by using the same protocol as before but after the excess of crosslinking solution had been wiped dry. The printability of each material was evaluated by flow behavior, filament size, pore size, grid uniformity, and patch height. This was performed by comparing the grid structure imaged directly after printing as well as after crosslinking occurred. Three patches of each ink type were evaluated—size estimates were performed using ImageJ 1.53t (NIH, Bethesda, MD, USA) at three random sites, respectively. The mean size (width) of the main filaments and pores were estimated while smaller pores along the contour were excluded.

### 2.4. Release Profile

HTX is a liquid with a complex composition rich in proteins. Proteins can easily be detected using small volumes, and release of proteins was therefore used as an indicator for the total release of HTX. The release of protein (HTX) from the crosslinked hydrogel structures was systematically studied starting with a solid disk when optimizing the ink formulation for which the protein release was analyzed using a BCA protein analysis kit, see Supplementary Materials for details about the method and results. Thereafter, the release from the selected R4 ink crosslinked solid disk was evaluated in different medium of increasing complexity including: 0.9% NaCl, phosphate buffered saline (PBS), chemically simulated wound fluid (CSWF), cell medium with 10% fetal bovine serum (DMEM-F), and FBS + peptone medium (FBSpept), see Supplementary Materials for details about the medium recipes, experimental method, and results. Finally, the protein release was evaluated for the selected R4 ink 3D printed hydrogel, Collex, where the manufacturing has been described in the 3D printing section. Collex (approx. 200 mg), stored in sachets for 1 to 4 weeks, was transferred individually to a 24-well plate and 1 mL of cell media (DMEM supplemented with 10% FBS and 1% Pen/Strep) was added. The antibiotics were added to match the release medium used in the bioactivity assay. The plate was incubated at 37 °C, 5% CO_2_, humidified atmosphere. At indicated time points, 2 µL cell media was removed and the protein concentration was determined using NanoDrop™ (One/One^C^, Thermo Scientific, Madison, WI, USA) at 280 nm.

### 2.5. Chemical Characterization

The extractions of Collex were performed according to ISO 10993-12:2021 in duplicate using 0.9% NaCl in Milli-Q water and isopropanol as extraction media with an extraction ratio of 0.2 g/mL, and 5–10 patches per extraction. Patches were cleaned of excess cross-linking solution by dipping in saline for < 1 s. The extractions were conducted at 50 °C for 72 ± 2 h.

Chemical analysis with gas chromatography–mass spectrometry (GC–MS) and inductively coupled plasma mass spectrometry (ICP–MS) was performed within 24 h after the extraction was ended. The extraction procedure was followed by determination of extracted organic and inorganic components with GC–MS and ICP–MS, respectively, according to ISO 10993-18:2020. Additional details about the extraction protocol can be found in the Supplementary Materials.

### 2.6. Bioburden

Evaluation of microbial cleanliness (bioburden) of Collex following the European Pharmacopeia 2.6.12: Microbial enumeration tests. The HTX solution 12% (*v*/*v*) and Collex was tested for any inhibitory (antimicrobial) effect on the growth of microorganisms, see Table 2, which was included in the test and to compensate for it if needs be. All samples were handled in a laminar air flow cabinet (LAF) with a high-efficiency particulate absorbing (HEPA) air filtration system.

HTX and Collex were evaluated for bioburden by total aerobic microbial count (TAMC) and for total yeast and mold count (TYMC). Both products were tested in triplicate. Sterility of the environment was evaluated in parallel by negative controls of tryptone soy broth (TSB), TSB-100, and 0.9% saline spread on TSB, tryptic soy agar (TSA) and sabouraud dextrose agar, (SDA), respectively. The methodology in full can be found in the Supplementary Materials.

### 2.7. Bioactivity and Biocompatibility

#### 2.7.1. Cytotoxicity

The cytotoxic potential of Collex was analyzed by extraction of the test item according to ISO 10993-12:2021 and cytotoxicity testing according to ISO 10993-5:2009 Annex C (MTT cytotoxicity test) in a GLP approved test facility.

#### 2.7.2. Cell Cultivation

Human fibroblast cell line, Hs 707 from ATCC (CRL-7449), was cultivated at 37 °C in a humidified atmosphere supplemented with 5% CO_2_. The cells were grown in Dulbeccos Modified Eagles Medium (Sigma-Aldrich, D0822) supplemented with 10% FBS (ATCC, 30-2025) and 1% Penicillin-Streptomycin (Sigma-Aldrich, P4333). For the pro-collagen I alpha 1 assay, cells at passage 2 to 5 were used.

#### 2.7.3. Pro-collagen I Alpha 1 Assay

Hs 707 cells were diluted to a concentration of 12 × 104 cell/mL and 1 mL was seeded onto 24 well plates and incubated in a cell incubator for 24 h. The following day (day 1) Collex (ca 200 mg) was transferred to cell media (1.2 mL) and placed in a cell incubator for 4 h for release of HTX. Absorbance at 280 nm was measured to ensure the protein concentration was the same as 1% stock solution of HTX or higher. On the cell plate, cell media (1 mL) was replaced with the following: new cell media, cell media with extract from Collex, cell media with extract from printed control (without HTX), or cell media with 1% HTX (stock solution). The cells were incubated further in a cell incubator and at day 4 and 6, new cell media was prepared as for day 1, but on these days, only 300 µL of the cell media was replaced. At day 8, cell media was collected from each treatment and the level of pro-collagen I was determined with human pro-collagen I alpha 1 ELISA kit (ab210966, Abcam, Cambridge, UK) according to manufacturer’s recommendations.

## 3. Results and Discussion

### 3.1. Ink Rheology

**Figure 1:**
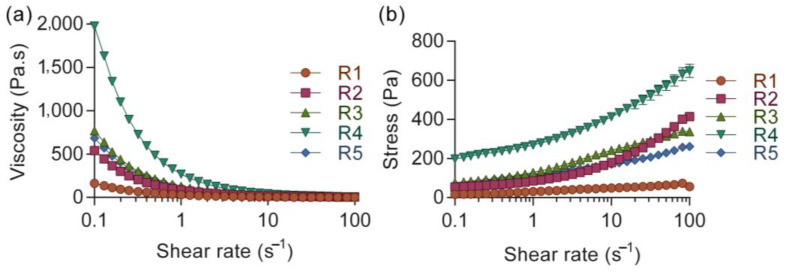
(**a**) Viscometry conducted on the bioinks, here shown with respect to viscosity (Pa·s) vs. shear rate (s^−1^), and (**b**) shear stress (Pa) vs. shear rate (s^−1^). Mean values presented with SD (n = 3).

Extrusion-based 3D printing is dependent on the rheological behavior of the ink to allow for printability through the syringe. To achieve acceptable printability, the ink needs to have shear thinning to flow through the nozzle when subjected to low forces [38,39]. Five ink formulations were characterized by viscometry to assess the printability and the flow of the ink formulations. In Figure 1a, the flow sweep shows that the viscosity of R1 was the lowest among the tested inks, with an initial viscosity near 20 Pa·s at a shear rate of 0.1 s^−1^. Additionally, R1 showed no distinct shear thinning ability—there was no decrease in viscosity for increasing shear rate, likely due to the low amount of both nanocellulose and alginate. This could result in difficulties to extrude the ink on the build plate in a controlled manner. The viscosities of R2, R3, and R5 at low shear were similar and could barely be distinguished from each other, see Figure 1a. The ratio of alginate and nanocellulose differed between R3 and R5, but seemed to balance each other in terms of viscosity, whilst R2, with a lower nanocellulose content, resulted in slightly reduced initial viscosity despite the increased alginate content, see Table 1. The R4 ink formulation contained the highest amount of both alginate and nanocellulose, which resulted in an increased viscosity at low shear as well as increased shear thinning behavior in comparison to the others, see Figure 1a. The viscosity of the ink increases with the polymer concentration and the shear thinning is likely to be explained by disentanglements of polymer chains when subjected to a high shear rate [40,41,42]. The combination of these characteristics is considered positive for printing in terms of extrudability.

In addition, the ink needs to have a sufficient yield stress to retain the shape of the printed structure on the build plate. A high shear thinning ability allows for an increased printing speed to be utilized whilst the yield stress is related to the ink’s ability to retain its geometry on the build plate [38,39]. Generally, an increased yield stress supports printing of overhang structures with minimal risk of collapse. To gain further information regarding the non-Newtonian behavior of the ink, the shear stress was fitted to a Herschel–Bulkley model, Figure 1b and Table 3, which disclosed that the yield stress of R4 was distinctly higher compared to the other inks. R1 showed the lowest yield stress value while R2, R3, and R5 were alike. R4 showed more than a three-fold increase in yield stress compared to the other inks, which is indicative of better shape retention once printed. The factorial model showed a significant positive interaction effect between alginate and nanocellulose as shown in the Pareto chart (Figure S1, Supplementary Materials).

**Figure 2:**
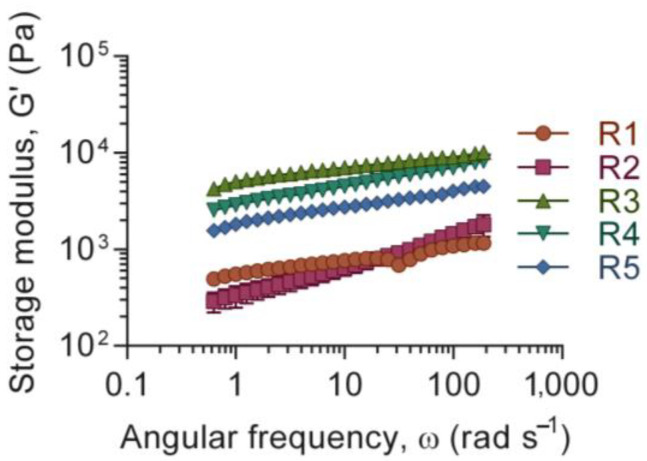
Frequency sweep describing the viscoelastic properties of the ink in terms of storage modulus, G’ (Pa), in relation to angular frequency, ω (rad s^−1^). Mean values presented with SD (n = 3).

Apart from the flow behavior, which describes the rheological behavior during printing and the ability of the ink to retain its shape on the build plate, the viscoelastic property of the ink describes additional characteristics of the formulation. In this study, the storage modulus (G’), evaluated by a frequency sweep, was plotted against the angular frequency, see Figure 2. It revealed that the ink formulations were similar with respect to angular frequency, typically related to the level of entanglement in the physical gel [43]. The tests were conducted before crosslinking of the alginate, and no cross-over point was seen, i.e., the ink displayed a gel-like appearance, within the frequency range tested (data not shown). The main contribution to G’ was nanocellulose concentration and no other modeling of the data was performed. R1 and R2 showed a similar absolute value of G’, lower in comparison to R3–R5. The samples R3–R5 displayed a similar range in terms of G’, most likely due to their higher content of nanocellulose, see Table 1. Nanocellulose is known to have a reinforcing ability that affects the viscoelastic properties of bioinks, which has shown to be favorable for printability [44]. The cellulose nanofibers can support the stability of the ink through their entanglements at low concentration [45].

### 3.2. Three-Dimensional Printing and Structural Characterization

**Figure 3:**
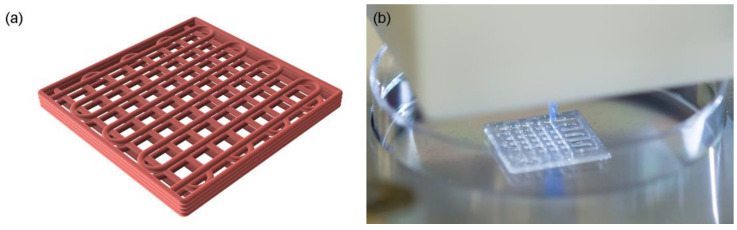
Schematic CAD drawing of the continuous interlaced grid design (**a**), and an ongoing print of the second layer using the R4 ink (**b**). The dimensions of the CAD grid were 16 mm × 16 mm × 1.37 mm with a pore size of 1.4 mm × 1.4 mm.

The inks listed in Table 1 were evaluated for printability and shape fidelity based on the resulting overall grid uniformity, filament width, pore size, and patch height. The printing parameters of each ink were individually optimized prior to the evaluation, where patches of four layers were printed in a predetermined design and compared to the template CAD drawing, see Figure 3a,b.

**Figure 4:**
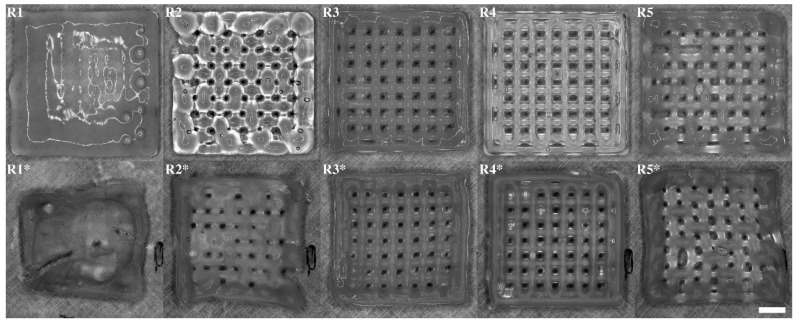
Representative 3D printed ink patches (four layers) imaged directly prior to (**R1**–**R5**) and after (R1*–R5*) crosslinking for >30 min. The white scale bar represents 3 mm.

The observations made during printing of inks R1–R5 agreed with the rheological results. A higher content of alginate, as in R2 and R4 compared to R1 and R3, respectively, resulted in improved printability and shape fidelity of the inks, see Figure 4. This is likely due to an increased viscosity, shear thinning behavior, and yield stress. Even though R2 and R3 showed similar shear thinning ability and yield stress levels, the R3 ink displayed an improved printability and shape retention most likely due to an increased G’. The R1 and R2 tended to drip during printing and showed low to non-existing shape retention, which can be correlated to their lower yield stress and G’, which was in line with the rheological observations where R3–R5 showed increased viscoelastic properties. To achieve a continuous flow through the needle, the required printing pressure increased with the ink number going from R1 to R4 due to its increased viscosity and viscoelastic behavior. R5 required a similar pressure to R3 but needed a much higher printing speed due to the increased flow through the syringe. The HTX content was also observed to slightly improve the printability when compared to an ink where the HTX was replaced by saline, as in the control patches used in some of the other experiments such as bioactivity testing. The HTX-containing ink R4 showed better shape retention than the corresponding ink without HTX but displayed a higher tendency of air entrapment and increased hydrophobicity. This is likely to be explained by the surface activity of proteins and polar lipids in HTX, which tend to be enriched at the interphase between water and air resulting in stabilization of bubbles and increased hydrophobicity. This was evident when the patches were exposed to the crosslinking solution, see Figure S2 in Supplementary Materials. The patches based on R1, R3, and R5 with a lower content of alginate appeared rather soft and sensitive, risking deformation when handled, apparent for R1* and R5* in Figure 4.

The patch characteristics for R1–R4, presented in Table 4, showed increasing grid uniformity, filament consistency, and conformity for increasing ink number compared to the CAD design (Figure 3a), as reflected by the visual results shown in Figure 4. The structural characteristics were in line with the rheological observations, where a combination of high shear thinning, yield stress, and G’ resulted in improved 3D printing properties, as in R4, and then decreased with decreasing ink number. All the inks showed dye swell, judged by the increased filament width in comparison to the nozzle size with an internal diameter of 0.41 mm, Table 4. The printing protocol included a 20% filament overlap between layers, targeting 1.37 mm in total height. The resulting patch height estimated after crosslinking of the structure ranged between 0.5 and 1.0 mm (see Table 4), indicative of some structural collapse, and flow as can be seen in Figure 4. The patches displayed a slight shrinkage when crosslinked (not quantified). The ink could most likely accomplish a denser lattice structure and perhaps been optimized further by changing the composition by, for example, increasing the biopolymer content or by additional additives. However, given the overall printability and characteristics in combination with a maintained structural stability during storage (patches that had been stored in solution for 4 weeks were used in the bioactivity assay in 3.6), the grid structure achieved by the R4 ink was determined suitable for the given application.

### 3.3. Release Profile

The release of HTX from the crosslinked hydrogel was systematically studied starting with a solid disk design to support the optimization of the ink formulation. The protein release appeared similar regardless of the hydrogel composition within the evaluated range, see Figure S3 in Supplementary Materials. Thereafter, the release was evaluated in release-media of varying complexity. The loading of HTX into the hydrogels is non-specific (non-covalent), and various types of physical molecular interactions are expected to influence the binding strength of the HTX components to the gel structure. Therefore, pH, ionic strength, and protein content may affect the release rate of HTX from the hydrogels. The release profile may thus vary depending on the composition of the surrounding medium. For wound healing applications, it is important that the bioactive ingredients are released into the wound exudate, which consists of a crude mixture of proteins, salts, nutrients, and inflammatory cells and components [46]. Saline, specifically 0.9% NaCl_(aq)_, was utilized as a release medium in order to establish a controlled system for release. Its purpose was not to mimic the actual wound environment, but as a controlled system for initial understanding and characterization without interactions with other medium components. Additionally, phosphate buffered saline (PBS) was used as a more physiologically relevant buffer. A recipe of a chemically simulated wound fluid (CSWF) was found in the literature [47]. The CSWF is composed of ions relevant to a wound environment as well as a protein, bovine serum albumin (BSA), in amounts that would correspond to the total protein content in a wound. It is a chemically defined medium, which offers a high level of control in in vitro assays. For the more complex protein-mixtures, fetal bovine serum (FBS) was added either in low amounts (10%) to cell culture medium with full nutritional value for cell culturing (DMEM-F), or in high amounts (50%) directly into peptone water (FBSpept); the latter is referred to as simulated wound fluid in the literature [48]. A substantial release of HTX occurs in both protein-free PBS and in protein-rich media, such as CSWF, DMEM-F, and FBSpept, see Figure S4 and Table S1 in Supplementary Materials. This is of importance since the hydrogel was designed to be used as a dressing applied directly on wounds, where the surrounding environment is rich in both proteins and other biological components, in which the serum has the highest resemblance with. The release results confirmed that constructs made from the R4 ink will release HTX into surrounding fluids that are similar to a real wound environment.

**Figure 5:**
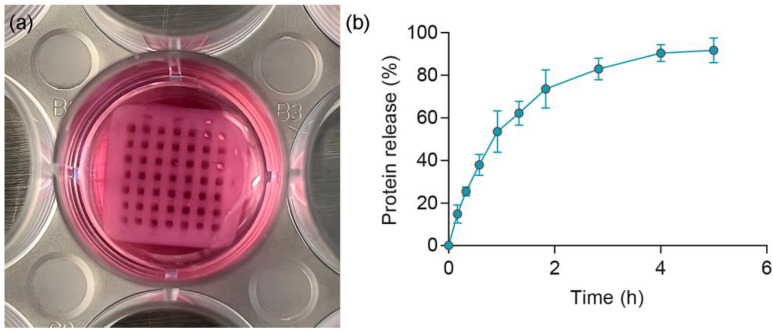
The patches (**a**) were incubated at 37 °C, 5% CO_2_, in a humidified atmosphere during the analysis. The absorbance of DMEM + FBS blank was subtracted from each measurement. Protein release of Collex in 1 mL DMEM supplemented with 10% FBS and 1% Pen/Strep determined by absorbance at 280 nm (**b**), n = 5 presented as a mean ± SD.

Finally, the release of HTX into a complex medium was studied for the selected hydrogel, Collex, 3D printed from R4 ink. Non-labelled HTX was used in the construction of 3D-printed Collex patches. Crosslinking was carried out in a CaCl_2_ solution with added HTX 12% (*v*/*v*), the same concentration as in the R4 ink, to avoid HTX release losses during the crosslinking step. A time-dependent release into DMEM-F was also observed for Collex. Total protein release from Collex patches into DMEM supplemented with 10% FBS and 1% Pen/Strep to match the medium used in the bioactivity assay is shown in Figure 5 a, b. The total protein content was measured using absorbance at 280 nm and the absorbance from the medium (DMEM-F with Pen/Strep) was subtracted for each time point. Compared to the solid disks, protein release from the 3D printed Collex patches was both faster and resulted in markedly higher total released amounts. Approximately 90% of the protein was released from the 3D-printed patch that had been crosslinked in CaCl_2_ solution containing HTX. It is possible that even higher levels can be released in a wound where protein exchange occurs, which shifts the equilibrium. There were several differences between the solid disk and printed patch experiments that may explain the difference in their release profiles. With printing, there is a more precise control of the patch size, and the patches were weighed after preparation to ensure consistency between samples. In addition, they were stored for 1 to 4 weeks in sachets with storage solution prior to use. The crosslinking solution as well as storage solutions contained added HTX to avoid release from the patch. However, one cannot exclude the possibility that the patch will absorb HTX from the surrounding environment during preparation and storage, resulting in a higher total amount of HTX in the samples than expected. If that is the case, the percentage of total protein that has been released calculated based on the amount of HTX in the ink prior to printing would overestimate the actual percentage of released protein. This would be interesting to investigate further, although it would be difficult to delineate any possible absorption during storage accurately. However, such assessment is not depicting the usefulness of this patch in wound care, as long as the storage conditions are well-defined, and the patch has the desired effect when used. An important factor influencing the release rate and total released amount is the structures and surface areas of the patches; the lattice structure has a larger surface area compared to the solid disks, which makes the distance the proteins must travel through the gel structure shorter before reaching the surrounding medium. It is difficult to obtain a detailed insight in the release mechanism by only measuring protein release by absorbance at 280 nm. However, it was important to study the behavior of non-labelled HTX to allow native interactions of HTX with the gel structure, as well as using a complex medium representing a wound environment, which in combination, constrained the choice of detection methods. The results showing an effective release are encouraging for further use of the Collex patch.

### 3.4. Chemical Characterization

The chemical components of Collex were characterized to assess its safety profile, limited to volatile and semi-volatile organic compounds as well as inorganic elements. The extract solutions from Collex, either saline or isopropanol, were transparent and uncolored with no visible particles. The hydrogel remained visibly unchanged after the extraction. The extracts were analyzed in triplicate. Leached organic compounds were screened by the GC–MS method, where mainly fatty acids, cholesterol, and saccharides (predominantly mannitol) were identified in the isopropanol extracts. Measured concentrations are considered non-toxic. Chromatograms and a complete list of organic compounds can be found in Figures S5 and S6, and Table S2 in the Supplementary Materials. Leached inorganic elements identified with ICP–MS in the saline extracts of Collex patches are presented in Table 5. The reason for using two different extract solutions, saline and isopropanol, was that inorganic elements have a much higher solubility in saline compared to isopropanol, while the opposite applies for organic compounds. This was why no organic compounds and no inorganic elements were identified in the saline and the isopropanol extracts, respectively. A complete list of all inorganic elements and aliquots analyzed can be found in Tables S3 and S4 in Supplementary Materials. The toxicological risk assessment following the chemical characterization revealed margins of safety that did not indicate any risk for acute systemic toxicity from using the Collex device. Moreover, the assessment revealed no leaching that is known to cause material-mediated pyrogenicity and concluded that leaching of compounds under clinical conditions is unlikely to cause skin irritation or sensitization.

Wound healing and nutrition are closely linked, where nutrient deficiency impedes the normal healing process by prolonging the inflammatory phase, and reduces fibroblast proliferation and collagen synthesis [49]. Iron homeostasis in the skin is important for the cutaneous wound healing process, where both iron deficiency and overload have shown negative effects [50]. Strontium salt has been shown to suppress TNF-α levels and thus inflammation levels [51], while both zinc and manganese have been shown to modulate the expression of integrins affecting, for example, the proliferation phase of wound healing [52]. Zinc supplements have been found to enhance wound healing, especially when administrated topically, where zinc ions stimulate epithelialization and reduce superinfections and the amount of necrotic material [53]. Additionally, zinc has been found effective specifically for healing of diabetic foot ulcers [54].

### 3.5. Bioburden Analysis

Bioburden analysis was performed to determine the sterility of the finished product. The suitability of test method and growth promotion test was successful, since the small amount (<100 CFU) of micro-organisms inoculated onto agar was recovered, see Table S5. The inoculation together with either the HTX solution or the Collex patch showed no inhibitory (antimicrobial) effect. Both the HTX solution and the Collex patch were found to be sterile with no growth found in any of the samples, indicating adequate routines during aseptic formulation, production, and packaging.

### 3.6. Bioactivity and Biocompatibility

**Figure 6:**
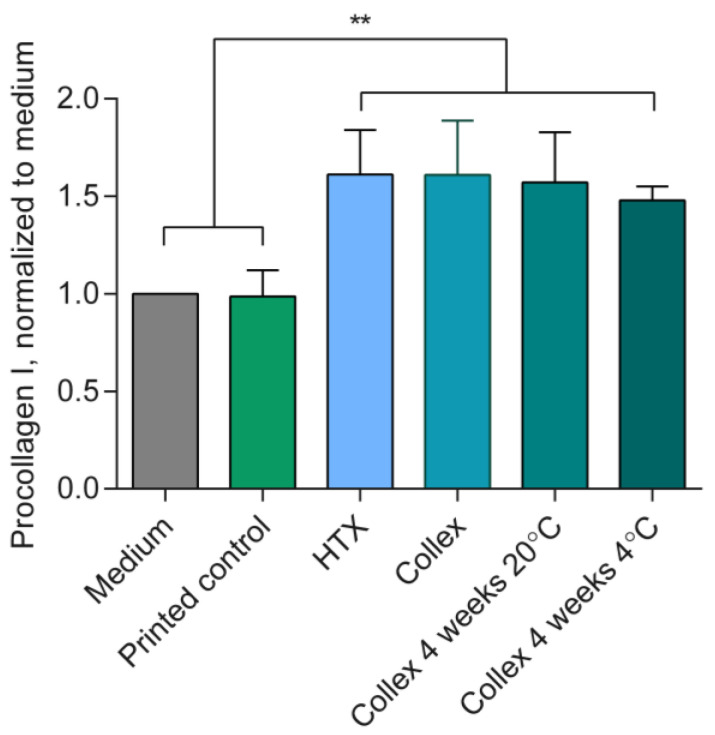
Pro-collagen I alpha 1 ELISA assay results normalized to medium control. Data presented as mean with SD; n = 4, except for Collex 4W 20 °C and Collex 4W 4 °C where n = 2; these were stored at room temperature and fridge for 4 weeks, respectively. ** Indicates significant difference (*p* < 0.01).

Collagens are converted from pro-collagens and play critical roles in wound healing [55]. Unpublished data from Regenics AS has shown that HTX stimulates the production of pro-collagen I alpha 1. For this reason, the bioactivity of released HTX was determined based on its ability to stimulate collagen production, measured by the pro-collagen I alpha 1 ELISA assay. Collex containing HTX resulted in significantly higher production of procollagen I alpha 1 compared to negative controls (*p* < 0.01). As expected, the R4 ink printed control without HTX did not affect pro-collagen I alpha 1 production. A maintained bioactivity was confirmed for Collex after storage for 4 weeks in a fridge as well as at room temperature, see Figure 6. A visual comparison between the Collex and the printed control patch can be seen in Figure S2, Supplementary Materials. In the release assay, only proteins were measured and the release of fatty acids and other compounds that may contribute to the effect of HTX were not detected. However, because results from the pro-collagen I alpha 1 assay showed that the increase in pro-collagen was similar for Collex and HTX, compounds contributing to the bioactive effect of HTX appear to be released. Collagen is reported to boost wound healing by accelerating formation of extracellular matrix, attracting fibroblasts, and act as an anti-inflammatory, and for these reasons, it is often used in wound dressings [56]. Collex does not contain collagen but appears to stimulate the native collagen production in fibroblast cells. If this stimulation can be extrapolated to wounds, Collex has great potential for enhancing wound healing. The extract of Collex was found non-cytotoxic when evaluated according to ISO 10993-5:2009. Results from a minipig trial [37] suggest that Collex has significant potential to improve healing of burn wounds and motivates further clinical testing of Collex in the healing of burns and chronic hard-to-heal wounds.

In summary, an all-marine ecologically sustainable bioactive wound dressing, Collex, was developed. A novel bioactive salmon roe extract, HTX, was introduced and loaded in the nanocellulose and alginate ink, and 3D printed to form a lattice structured hydrogel. The 3D printed hydrogel showed suitable mechanical properties enabling handling. It is likely that the design can be optimized further to tailor the release kinetics to the size and severity of the wound. There are currently several promising concepts under development involving polymers and additives for enhancing wound healing [16,24,57]. We present a composition that can be 3D printed and releases an active component with a retained bioactivity enhancing synthesis of ECM components. Moreover, it is advantageous to realize a sustainable solution moving towards a circular economy. Collex was found to be biocompatible with no toxicological risks identified and presents positive results that motivates further testing. The treatment of chronic hard-to-heal wounds looks promising, and it will be interesting to see what the future holds based on the current trends and ongoing research for new wound dressing concepts [57].

## 4. Conclusions

With components solely from marine sources, a 3D printable ink was successfully formulated with alginate, nanocellulose, and the unfertilized salmon roe extract HTX. The ink was used to make solid hydrogel disks as well as 3D printed lattice structures. The ink showed suitable printability and fidelity to realize uniform lattice structures, which is suggested as the major factor facilitating protein release of the printed structures compared to non-printed solid disks of the same formulation. The release of total protein content from hydrogels, representing HTX release, was studied during 24 h, and the release profile was favorable in the different types of simulated wound fluids with complex and protein-rich compositions, compared to regular saline solution. The HTX released from the final patch design, Collex, into cell medium containing physiologically relevant salts and nutrients as well as 10% FBS, enhanced pro-collagen I alpha 1 production in cell culture to the same level as the positive control with HTX in solution. A retained bioactivity was seen for Collex that had been stored in room temperature for 4 weeks as well as in the fridge. Collex was found to be non-cytotoxic and the toxicological risk assessment following the chemical characterization revealed margins of safety that did not indicate any risk for acute systemic toxicity or material-mediated pyrogenicity from using the Collex device. Collex was also concluded to be unlikely to cause skin irritation or sensitization under clinical conditions.

## Figures and Tables

**Table 1:** The composition of the evaluated ink variations presented as percentage (*v*/*v*).

Ink	Alginate (%)	Nanocellulose (%)	HTX (%)
R1	0.5	1.0	12
R2	2.0	1.0	12
R3	0.5	1.7	12
R4	2.0	1.7	12
R5	1.0	1.4	12

**Table 2:** Micro-organisms included in the evaluation.

Micro-Organisms	American Type Culture Collection (ATCC) Number
*Staphylococcus aureus*	6538
*Pseudomonas aeruginosa*	9027
*Bacillus subtilis*	6633
*Candida albicans*	10,231
*Aspergillus brasiliensis*	16,404

**Table 3:** Calculated yield stresses presented with SD for the different ink compositions.

Ink	Alginate (%)	Nanocellulose (%)	HTX (%)	Yield Stress (Pa)
R1	0.5	1.0	12	6.7 ± 3.1
R2	2.0	1.0	12	34.7 ± 3
R3	0.5	1.7	12	28.8 ± 1.9
R4	2.0	1.7	12	116.2 ± 6
R5	1.0	1.4	12	33.9 ± 5.3

**Table 4:** Characterization of the ink’s printability and shape fidelity, presented as mean values ± SD, n = 3 for patch height (crosslinked) and n = 9 for filaments and pores (non-crosslinked).

Ink	Grid Uniformity	Filament Width (mm)	Pore Size (mm)	Patch Height (mm)
R1	Non-existing	N/A	N/A	0.5
R2	Low	0.98 ± 0.19	0.77 ± 0.19	0.7
R3	Middle	0.65 ± 0.08	1.19 ± 0.12	0.9
R4	High	0.54 ± 0.05	1.24 ± 0.06	1.0
R5	Low	0.83 ± 0.25	0.94 ± 0.25	0.7

**Table 5:** The main elements and their amount identified with ICP–MS in saline extracts of the R4 ink hydrogel. The extraction was performed on duplicates (a, b), and the extracts were analyzed in triplicate presented as mean values ± SD, n = 3.

Element	Amount (ng/g Test Item (a))	Amount (ng/g Test Item (b))
Iron, Fe	510 ± 23	530 ± 0
Manganese, Mn	430 ± 25	470 ± 6
Strontium, Sr	210 ± 6	230 ± 0
Zinc, Zn	3300 ± 58	3600 ± 58

## Data Availability

See Supplementary Materials for supporting data.

## Supplementary Materials

The following supporting information can be downloaded at: https://www.mdpi.com/article/10.3390/polym15122627/s1, Additional experimental details, Figure S1: Pareto chart of standardized effects for the yield stress variable, Figure S2: Printed Collex and control patch, Figure S3: Protein release for solid disks of various composition, Figure S4: Protein release for solid disk in various release media, Figures S5 and S6: GC chromatograms, Table S1: Positive controls in the protein release experiment, Table S2: Hydrogel isopropanol extracts GC–MS analysis, Tables S3 and S4: Hydrogel isopropanol and saline extracts ICP–MS analysis, Table S5: Suitability of test method and growth promotion test results.
