# Supplementary material for: Development of an All-Marine 3D Printed Bioactive Hydrogel Dressing for Treatment of Hard-to-Heal Wounds
Source: Polymers (Basel). 2023 Jun 9;15(12):2627. doi: 10.3390/polym15122627 (PMC10301934; doi:10.3390/polym15122627)
Supplement: Supplementary file 1 [file polymers-15-02627-s001.zip › polymers-2401294-supplementary.pdf]

polymers

MDPI

Supplementary Materials

Development of an All-Marine 3D Printed Bioactive Hydrogel Dressing for Treatment of Hard-to-Heal Wounds

Patrik Stenlund [1,*], Linnea Enstedt [2], Karin Margaretha Gilljam [3], Simon Standoft [1], Astrid Ahlinder [4], Maria Lundin Johnson [2], Henrik Lund [3], Anna Millqvist Fureby [2] and Mattias Berglin [1,5]

[1] Department of Methodology, Textile and Medical Technology, RISE Research Institutes of Sweden AB, Arvid Wallgrens backe 20, SE-413 46 Göteborg, Sweden
[2] Department of Chemical Process and Pharmaceutical Development, RISE Research Institutes of Sweden AB, Drottning Kristinas väg 61B, SE-114 28 Stockholm, Sweden; maria.lundinjohnson@gmail.com (M.L.J.)
[3] Regenics AS, Gaustadalléen 21, N-0349 Oslo, Norway
[4] Department of Agriculture and Food, RISE Research Institutes of Sweden AB, Frans Perssons väg 6, SE-412 76 Göteborg, Sweden; astrid.ahlinder@ri.se
[5] Department of Chemistry and Molecular Biology, University of Gothenburg, Kemigården 4, SE-412 96, Gothenburg, Sweden
* Correspondence: patrik.stenlund@ri.se; Tel.: +46-10-516-58-30

Materials and Methods

Release Profile for Crosslinked Solid Disks in Saline

To evaluate the crosslinked ink's release profile, single sample small circular disks (appr. 180 mg) were prepared, crosslinked in CaCl2 (500 µL), and washed in 0.9% NaCl (500 µL) in the lid of Eppendorf tubes (1.5 mL) measuring approximately 8 × 5 mm (D × H). Each step involved exposing the disk to each solution three times by turning of the tube. The protein release testing was performed in 1 mL 0.9% NaCl while the hydrogel was still in the lid, i.e., only exposing the top surface. The protein release was measured by sampling 100 µl and replacing the amount by NaCl (0.9%) at several time points (15, 30, 60, 90, 180, 960, 1020 min). The released total protein amount was analyzed in duplicates by a BCA protein analysis kit after 2 h incubation time in room temperature using a BioTek Synergy HTX multi-mode plate reader (BioTek Instruments, VT, US) at 562 nm. HTX was used to derive the standard curve within the range of 1 – 500 µg/ml instead of BSA.

Release Profile for the R4 ink Crosslinked Solid Disks in Different Release Media

Once the final ink composition was selected, additional release kinetic tests were performed. The release of HTX from R4 ink crosslinked solid disk over time was studied in five different medium types, with various compositions, corresponding to several levels of complexity as described herein starting with 0.9% NaCl, and phosphate buffered saline (PBS, Sigma Aldrich). A chemically simulated wound fluid (CSWF) according to the following recipe: 5.844 g/L NaCl, 0.2982 g/L KCl, 3.360 g/L NaHCO3, 0.2775 g/L $CaCl_2$ and 33 g/L BSA [33]. A cell medium with 10% fetal bovine serum (FBS), DMEM-F, prepared by adding FBS (Gibco) to Dulbecco's Modified Eagle Medium (Gibco, DMEM high glucose GlutaMAX Supplement pyruvate). Finally, a FBS + peptone medium, FBSpept, consisting of 50% FBS and 50% peptone water prepared by 0.1% peptone (Sigma Aldrich, peptone from soybean, enzymatic digest) in 0.9% NaCl.

Fluorescamine (FA, Sigma Aldrich, prepared as 5 mg/mL in acetone) was added to HTX to obtain 10% FA (v/v). Alginate, nanocellulose and FA-labelled HTX were mixed to the R4 composition. The mixture was transferred to a syringe and solid disk hydrogels were prepared by manual extrusion of 150 µL samples in a 24-well plate. Then 750 µL

crosslinking solution (0.1 M $CaCl_2$ in 0.9% NaCl) was added to each sample. After 15 min, the liquid was removed, and each disk was washed rapidly in 750 µl 0.9% NaCl. Thereafter, 1 mL release medium was added to each well. Duplicates was used for all release media except for PBS. Each medium without the hydrogel was included as negative controls while FA-labelled HTX in corresponding amounts was used as positive controls. Fluorescence was measured after 45 min, 2.5 h, 20 h and 24 h. For each measurement, liquid was moved from each well into a Nunclon Delta surface black 96-well plate (Thermo Scientific, Waltham, MA, US) and analyzed using a Varioskan Lux plate reader (Thermo Scientific, Waltham, MA, US) at $\lambda_{excitation}$ = 395 nm and $\lambda_{emission}$ = 477 nm. All samples were returned to the corresponding well after each measurement, so that the total volume was kept constant not to influence the release rate of HTX by changing the concentration equilibrium. The fluorescence was also measured in the crosslinking and wash solutions from each sample to identify any FA-labelled HTX that was lost from the hydrogel disks during sample preparation. These values were used to calculate a corrected total amount of HTX in each sample and the percentage released HTX in the release experiment, as follows. Positive controls were prepared from FA-labelled HTX in each medium type, and fluorescence was measured for the controls at the four time points. For each time point, the magnitude of fluorescence corresponding to 100% release of labelled HTX in each hydrogel sample was calculated by subtracting the fluorescence from lost FA+HTX in the crosslinking and wash solutions for a specific hydrogel sample from the fluorescence of the positive control in the corresponding medium. By doing this separately for each positive control measurement (in different medium and at different time points), any inhibition by medium components or photobleaching were considered when calculating the theoretical maximum fluorescence (TMF) upon 100% release of HTX from each hydrogel sample. The percentage released HTX at each time point of the release experiment was calculated from the fluorescence for each sample and the TMF at the corresponding time point. After this, the average percent of released HTX was calculated for duplicate samples.

Chemical Analysis

Besides cleaned and exposed to 50 °C for 72 ± 2 h, the vessels were continuously agitated at 30 rpm during extraction. Extracts for GC-MS analysis were stored in clean borosilicate glass containers until analysis. Extracts for ICP-MS analysis were diluted with acidified water in clean polypropylene centrifuge tubes immediately after that the extraction was ended and stored at room temperature until analysis.

For the GC-MS analysis, an internal standard Bis(2-ethylhexyl) phthalate (DEHP-d4) was added to the extracts and organic compounds eluting in the temperature range 100 °C – 350 °C determined, expressed as equivalents of DEHP-d4. The isopropanol sample was injected directly on the GC-MS, while the saline sample was extracted with n-hexane injection. The organic compounds were identified by their mass spectra by comparison to a reference library (NIST, version 2.2, 2014).

Bioburden Analysis

Suitability of Test Method and Growth Promotion Test

To establish the suitability of test method the samples were tested in presence of a small number of micro-organisms. The micro-organisms included in the study are presented in Table 2. Additionally, the growth promotion of the media and agar used in the test was established.

Overnight cultures of *S. aureus*, *P. aeruginosa* and B. *subtilis* were grown in TSB at 35 °C during dynamic conditions, while *C. albicans* was grown for 48 h at 25 °C. *A. brasiliensis* was grown on TSA until a dark thick film was formed and sporulation had occurred. Directly prior to use, spores were extracted from the agar plate by adding 1 mL TSB and rubbing the surface with an inoculation loop. Spores were collected with a pipette and added to a 50 mL centrifugation tube. All microbial suspensions were centrifuged at 2,000 × g, supernatant removed, and pellet resuspended in 1 mL TSB diluted 100 times in MQ water (TSB-100). A 1:2 dilution series was made, and optical density (OD) measured.

From OD 0.1 further dilutions were made to generate an inoculum with ≤ 100 colony forming units (CFUs) per 10 μL.

In a LAF cabinet, the test items were inoculated with 10 μL of the microbial species, while the same amount of each species were spread on TSA for live count. *C. albicans* and *B. brasiliensis* were also spread on SDA. Extraction was performed by placing each test item in a 50 mL centrifugation tubes, pre-filled with 20 mL 0.9% saline. All tubes were vigorously vortexed for 1 min. The Collex patches were treated in an ultrasonic bath, using a frequency of 35-45 kHz and minimum power of 150 W, and vortexed again for 1 min. The extractants were filtrated and the filters placed on TSA agar plate for growth tests with *S. aureus*, *P. aeruginosa* and B. *subtilis* at 35 °C overnight, and *C. albicans* and *B. brasiliensis* at 25 °C for 48 h. The numbers of CFU found on the agar plates and filters were then compared to assess the suitability of the extraction method and growth on TSA and SDA, respectively.

Negative Controls

100 μl of TSB, TSB-100, and 0.9% Saline were spread on TSA and SDA, respectively. Agar plates (TSA and SDA) were also placed in the LAF cabinet during the test. All plates were incubated for 7 days at 30 °C for TSA plates and at room temperature for SDA plates, as control of sterility.

Bioburden Evaluation

In a LAF cabinet, the package with the test item was opened and transferred to a 50 mL centrifugation tube pre-filled with 20 mL 0.9% saline using a sterile tweezer. Both products, Collex and the HTX solution 12% (v/v), were tested in triplicates. All tubes were vigorously vortexed for 1 min. The Collex was also treated in an ultrasonic bath, using a frequency of 35-45 kHz and minimum power of 150 W, and vortexed again for 1 min, as described above.

The extractant solution from each test sample was then divided in two equal parts, one for total aerobic microbial count (TAMC) and one for total yeast and mold count (TYMC). Each part was filtered, and the filters were placed on TSA and SDA for TAMC and TYMC, respectively. TSA plates were incubated at 30°C for three days and SDA plates at room temperature for 5 days. The plates were inspected for growth on the first, third and fifth day.

Results

Full factorial design response

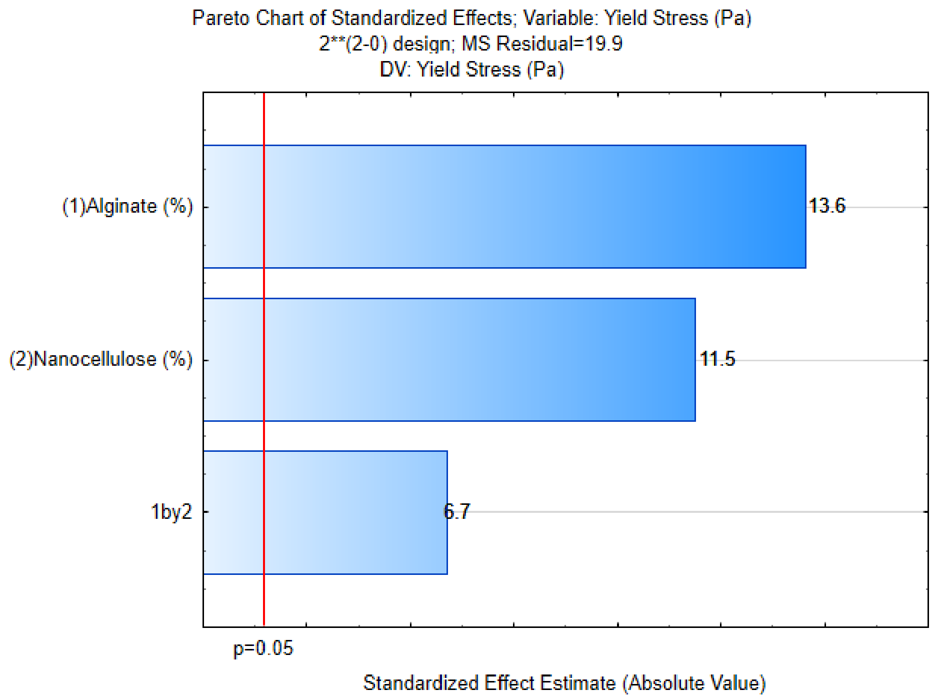

Figure S1. Pareto chart of standardized effects for the yield stress variable.

Patch images

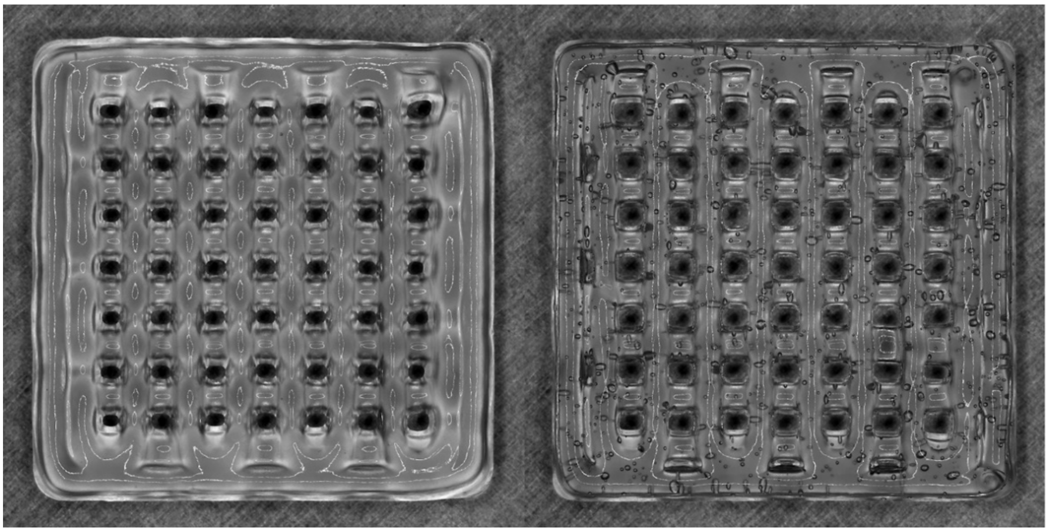

Figure S2. The finalized 3D-printed grid structure of the printed control without HTX (left) and Collex (Right).

Release kinetics

Release Profile for Crosslinked Solid Disks in Saline

Regardless of the hydrogel composition within the evaluated range, the release profile appeared robust, i.e., similar for the five formulations, as shown in Figure S3. The HTX recovery levels were low, which likely is a result of a combination of factors. First, the crosslinking and washing steps were performed in NaCl solutions without added HTX, which likely resulted in leaching of the total protein amount during these steps (not measured). Second, the solid design constitutes a relatively small surface area that is exposed to the release medium, which is of a limited volume. Both these aspects affect the equilibrium between protein in solution and protein remaining in the disks.

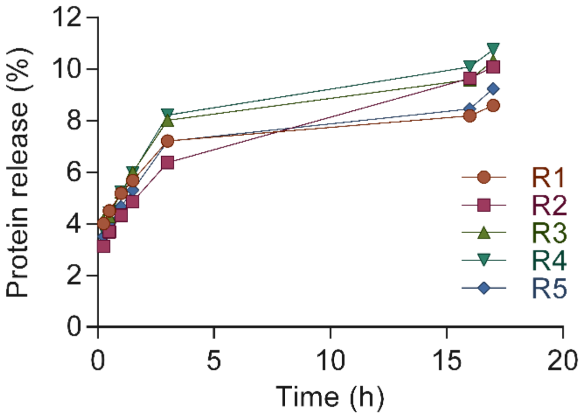

Figure S3. The released total protein amount of small solid hydrogels with varied ink composition determined by BCA analysis kit.

Release Profile of the R4 Ink Crosslinked Solid Disks in Different Release Media

The release of protein over time from solid disks of crosslinked R4 ink, see Figure S4a, was studied in different media types, with various compositions corresponding to several levels of complexity. In such complex protein mixtures, it is not possible to identify HTX components by using non-specific methods to detect proteins. Therefore, the HTX was pre-labelled with the fluorophore Fluorescamine that binds non-specifically to amine groups in all proteins in the HTX sample. In this way, the proteins of HTX can be detected by fluorescence in a non-labelled protein-rich media. Fluorescamine does only exhibit fluorescence after conjugation to proteins and not by itself, i.e., a low background fluorescence is to be expected. The fluorophore conjugation was evaluate, data confirming that the release results were not disturbed by free Fluorescamine can be found in Table S1. Release of HTX in five different media with various levels of component complexity was evaluated, see Figure S4b. The release was studied up to 24 h after the experiment was started. After considering the amount of HTX that were lost during preparation (crosslinking and washing) of each hydrogel, the percentage released HTX was calculated in relation to the corrected maximum amount of HTX in each sample.

Less HTX was released in NaCl than in all the other medium types. The reason for the lower release in saline compared to the more complex media remains uncertain but pH differences may have influenced the results. One hypothesis worth mentioning is that the large number of various components in complex media exhibit more sites to which the HTX components may find favorable interactions and bind to. That is, in a complex media, this may serve as a driving force for HTX components to leave the hydrogels and remain in the surrounding solution.

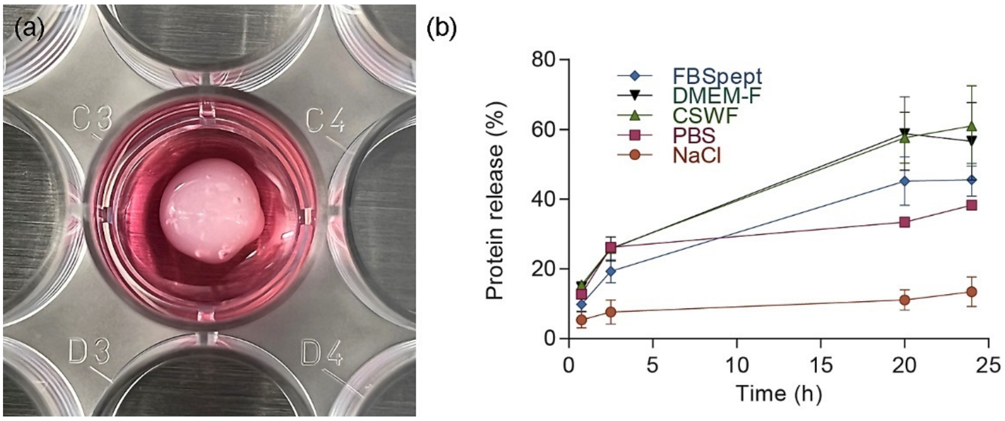

Figure S4. A solid disk from R4 ink in DMEM-F (**a**) and the release of HTX in 1 ml of NaCl, PBS, CSWF, DMEM+10%FBS (DMEM-F), and 50% FBS in peptone water (FBSpept), respectively (**b**). Experiment has been done once, with duplicate samples per medium, except for PBS. Respective

blanks have been subtracted from each data point. Mean values and standard deviations are shown, evaluated with regards to release of HTX in relation to corrected maximum fluorescence, in different media.

Table S1. Positive controls in release profile experiment at 1.5 h.

Sample	Medium	Fluorescence (a.u.)
HTX+FA in solution	NaCl	198
HTX+FA in solution	PBS	195
HTX+FA in solution	CSWF	202
HTX+FA in solution	DMEM	164
HTX+FA in solution	FBSpept	172

The fluorescence was not higher in the media containing proteins (CSWF, DMEM, FBSpept) after addition of a solution in which HTX and FA had been mixed, compared to protein-free media (NaCl, PBS). This indicates that all FA had been crosslinked to HTX prior to addition to media, so that no FA was free in solution and able to bind to proteins in the medium. Therefore, “false” fluorescence caused by FA binding to medium components is not likely to have contributed to the results in the release kinetics experiment.

Chemical characterization of Collex

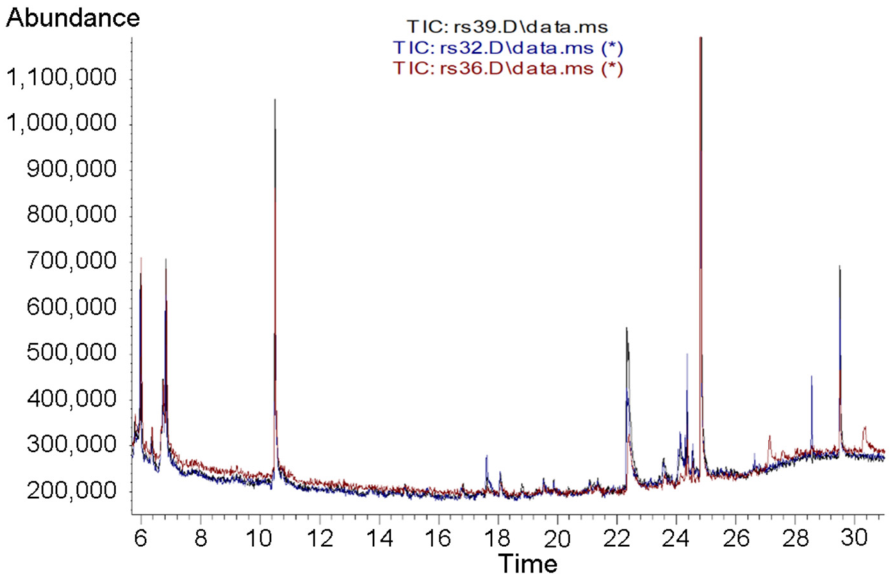

Figure S5. Representative GC-MS TIC (Total ion count) chromatograms for duplicate saline extracts (RS32 and RS36) of Collex as well as a blank (RS39). The highest peak represents the internal standard DEHP-d4.

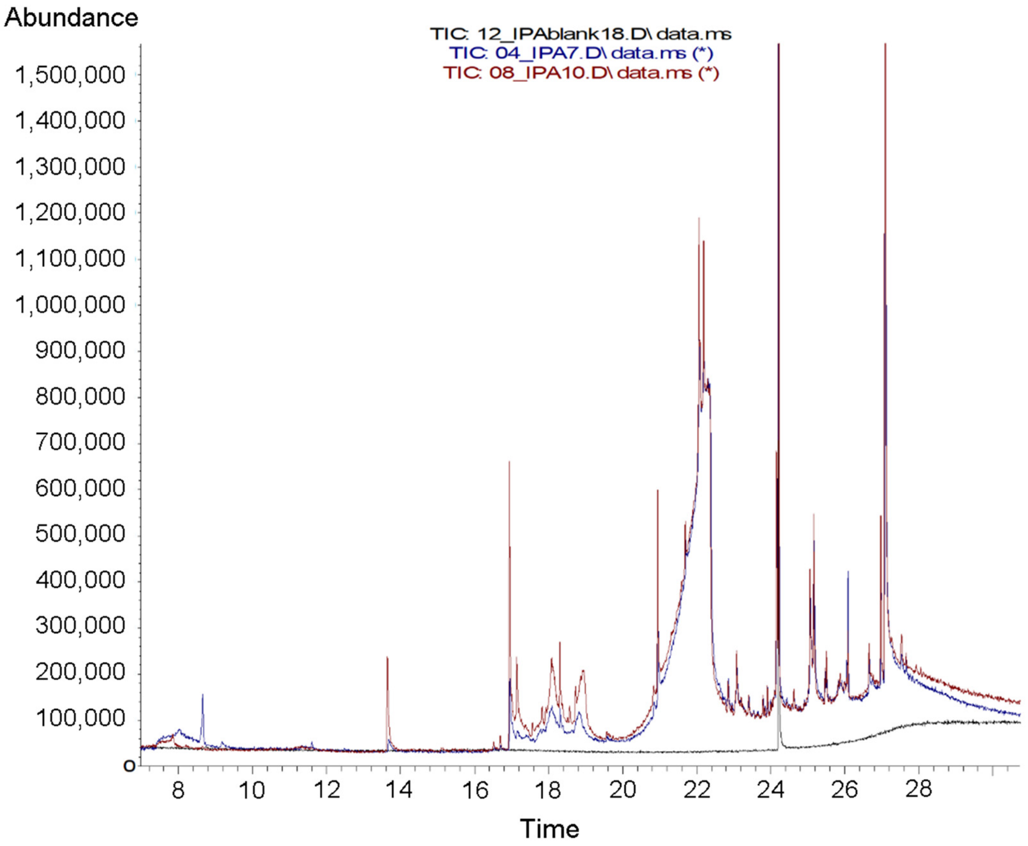

Figure S6. Representative GC-MS TIC (Total ion count) chromatograms for duplicate isopropanol extracts (04_IPA7 and 08_IPA10) of Collex as well as a blank (12_IPAblank18). The highest peak represents the internal standard DEHP-d4.

Table S2. Individual results of aliquots of isopropanol extracts of Collex analyzed by GC-MS.

		µg/g test item c[1]			µg/g test item d[1]		
**Compound**	**CAS#**	**Aliquot 1**	**Aliquot 2**	**Aliquot 3**	**Aliquot 1**	**Aliquot 2**	**Aliquot 3**
Isosorbide or possibly Dianhydromannitol	652-67-5	3.0	11	11	2.5	12	18
Unknown	-	11	20	26	15	34	36
Tetradecanoic acid or similar	544-63-8	8.7	13	15	11	12	15
Mannitol or similar	69-65-8	439	524	582	485	535	550
Hexadecanoic acid, 2,3-dihydroxypropyl ester	542-44-9	14	27	27	18	20	19
9-Octadecenoic acid (Z)-, 2,3-dihydroxypropyl ester	111-03-5	11	14	8.6	10	10	11
Octadecanoic acid, 2-hydroxy-1-(hydroxymethyl) ethyl ester or similar	-	13	20	18	10	13	11
Cholesteryl alcohol or similar	57-88-5	171	178	192	195	211	218

[1] The extraction was performed on duplicate test items, and the extracts were analyzed in triplicate.

Table S3. A complete list of the elements analyzed with ICP-MS in saline and isopropanol extract of Collex, respectively. Mean values adjusted to blanks is given within brackets for the elements above the limit.

	Saline extract		Isopropanol extract	
**Element**	**ng/g test item a[1]**	**ng/g test item b[1]**	**ng/g test item c[1]**	**ng/g test item d[1]**
Antimony, Sb	<75	<75	<75	<75
Arsenic, As	<500	<500	<500	<500
Barium, Ba	<75	<75	<75	<75

Beryllium, Be	<75	<75	<75	<75
Bismuth, Bi	<75	<75	<75	<75
Cadmium, Cd	<75	<75	<75	<75
Chromium, Cr	<75	<75	<75	<75
Cobalt, Co	<75	<75	<75	<75
Copper, Cu	<75	<75	<75	<75
Iron, Fe	510 (300)	530 (300)	<300	<300
Lead, Pb	<75	<75	<75	<75
Manganese, Mn	430 (75)	470 (75)	<75	<75
Molybdenum, Mo	<75	<75	<75	<75
Nickel, Ni	<75	<75	<75	<75
Silver, Ag	<75	<75	<75	<75
Strontium, Sr	210 (75)	230 (75)	<75	<75
Thallium, Tl	<75	<75	<75	<75
Tin, Sn	<75	<75	<75	<75
Titanium, Ti	<75	<75	<75	<75
Vanadium, V	<75	<75	<75	<75
Zinc, Zn	3300 (300)	3600 (300)	<300	<300

[1] The extraction was performed on duplicates, and the extracts were analyzed in triplicate. Presented values are the mean of the triplicate analysis of each extract.

Table S4. Individual results of aliquots of saline extracts of Collex.

	ng/g test item a[1]			ng/g test item b[1]		
**Element**	**Aliquot 1**	**Aliquot 2**	**Aliquot 3**	**Aliquot 1**	**Aliquot 2**	**Aliquot 3**
Iron, Fe	500	500	540	530	530	530
Manganese, Mn	410	440	460	470	470	480
Strontium, Sr	200	210	210	230	230	230
Zinc, Zn	3300	3300	3400	3500	3600	3600

[1] The extraction was performed on duplicate test items, and the extracts were analyzed in triplicate.

Suitability of the bioburden test method

Table S5. Results from suitability of test method and growth promotion test. Number of CFU found after inoculation with 10 µl on agar and number of CFU extracted after tested together with 12% HTX solution and Collex, respectively. CFU presented as average values of triplicate samples.

	HTX solution (12%)			Collex		
**Microbial specie**	**TSA**	**SDA**	**Filter (Bioburden)**	**TSA**	**SDA**	**Filter (Bioburden)**
S. aureus	69	NA	70	85	NA	56
P. aeruginosa	49	NA	76	58	NA	75
B. subtilis	43	NA	54	81	NA	85
C. albicans	80	85	94	92	101	104
A. brasiliensis	*	*	*	*	*	*

*Colonies grown together, counting not possible.
